# Oral belzutifan treatment in von hippel-lindau-associated retinal hemangioblastoma: a promising approach

**DOI:** 10.1186/s40942-026-00932-0

**Published:** 2026-09-14

**Authors:** Adnan Kilani, Abdelrahman Assaf, Constantin Jochem, Denise Vogt, Mona Laible, Melih Parlak, Wesam Alkabouni, Armin Wolf

**Affiliations:** 1 Department of Ophthalmology, University Hospital Ulm, Prittwitzstraße 43, 89075 Ulm, Germany; 2 Department of Neurology, University Hospital Ulm, Oberer Eselsberg 45, 89081 Ulm, Germany

**Keywords:** Von hippel-lindau disease, Retinal hemangioblastoma, Belzutifan, HIF-2α inhibition, Ocular oncology, Ultra-widefield imaging, Optical coherence tomography, Retinal tumors

## Abstract

**Background:**

Von Hippel-Lindau (VHL)-associated retinal hemangioblastomas (RHs) may cause vision-threatening exudation and hemorrhage. Conventional local therapies may be limited in advanced, recurrent, or anatomically high-risk lesions. This study evaluated ocular outcomes and observed tolerability during oral belzutifan treatment in a real-world cohort.

**Methods:**

Between January 2023 and June 2025, this retrospective observational study included five consecutive patients with genetically confirmed VHL, contributing seven eyes; each eye had at least one RH that progressed despite prior conventional local therapy. Patients received oral belzutifan 120 mg once daily. Median on-treatment follow-up (FU) was 43 weeks (IQR, 27–50); median documented cumulative belzutifan exposure was 43 weeks (range, 27–50). Data were available through January 2026. Tumor regression was quantified as the relative change in projected pixel area from baseline to last on-treatment FU. The largest baseline lesion per fundus-imaged eye was predefined for the primary analysis; all fundus-imaged lesions were assessed exploratorily. Fourteen fundus-imaged RHs were included in the projected-pixel-area analyses, and one MRI-assessed RH was reported separately. Three retinal specialists independently performed lesion measurements and exploratory overall ocular response (OOR) grading as separate assessments. Readers were masked to one another’s assessments; quantitative lesion measurements were additionally masked to patient information, treatment course, and image chronology. Safety was monitored within an interdisciplinary clinical framework.

**Results:**

In the predefined primary descriptive analysis of the largest baseline lesion per fundus-imaged eye (six eyes from four patients), all six index lesions regressed (median projected-pixel-area reduction, 58.3%; IQR, 17.2–64.7%). All four lesions in the patient-level sensitivity analysis regressed (median, 59.6%; range, 14.5–64.8%). Across all 14 fundus-imaged lesions, median regression was 57.2% (IQR, 28.5–66.9%), and 11/14 lesions (78.6%) showed > 20% regression. All three readers classified all six evaluable eyes as improved. Exudative retinal detachment resolved in three of four affected eyes. Drug-related adverse events were mostly mild to moderate and manageable.

**Conclusions:**

Oral belzutifan was associated with RH regression and improved exudative disease activity in this selected real-world cohort. These findings support systemic HIF-2α inhibition as a potential adjunctive therapeutic option for selected patients with progressive or anatomically high-risk ocular VHL disease. Prospective validation is required.

**Trial registration:**

not applicable.

**Clinical trial number:**

Not applicable.

## Introduction

Von Hippel-Lindau (VHL) disease is a rare autosomal-dominant tumor syndrome caused by pathogenic variants in the VHL tumor suppressor gene, resulting in constitutive activation of hypoxia-inducible factor (HIF) signaling and the development of highly vascular neoplasms across multiple organ systems, including the retina [1–4]. Retinal hemangioblastomas (RHs) represent the most frequent ocular manifestation of VHL disease, occurring in approximately 40–60% of affected individuals, and may lead to visual impairment due to progressive exudation, tumor hemorrhage, or exudative retinal detachment (RD) [5–8].

Management of VHL-associated RHs has traditionally relied on local ablative treatment modalities, including laser photocoagulation, cryotherapy, photodynamic therapy (PDT), radiotherapy, or vitreoretinal surgery [1, 3, 5–7, 9]. While small peripheral lesions may respond favorably, outcomes in advanced, posterior, or juxtapapillary RHs (JRHs) remain limited. In particular, PDT and other ablative approaches near the optic nerve head are associated with an increased risk of collateral retinal or optic nerve damage, vascular occlusion, persistent exudation, and irreversible visual loss [7, 10, 11]. Moreover, given the systemic and frequently bilateral nature of VHL disease, patients often face progressive involvement of both eyes over time, leaving limited visual reserve in advanced stages.

At the molecular level, loss of functional VHL protein disrupts oxygen-dependent degradation of HIF-α subunits, leading to sustained activation of hypoxia-responsive pathways. Histopathologic studies have demonstrated upregulation of HIF-1α and HIF-2α in VHL-associated RHs, accompanied by increased angiogenesis, metabolic reprogramming, inflammatory cell infiltration, and autophagic activity, particularly in lesions refractory to anti-vascular endothelial growth factor (VEGF) therapy [12]. As VEGF represents only one downstream effector of HIF signaling, VEGF inhibition alone may fail to achieve durable disease control, supporting the rationale for upstream targeting of HIF-2α.

Belzutifan is a selective oral HIF-2α inhibitor approved for the treatment of VHL-associated renal cell carcinoma (RCC), central nervous system (CNS) hemangioblastomas, and pancreatic neuroendocrine tumors. In the phase 2 LITESPARK-004 trial, belzutifan demonstrated clinically meaningful and sustained responses across systemic VHL manifestations, including consistent regression of ocular RHs in a dedicated subgroup analysis [13]. Published real-world evidence is no longer limited to isolated case reports. Jamshidi et al. described an institutional series of seven patients (12 eyes); Ercanbrack et al. reported a three-patient case series with Optos-based surface-area measurements; and single-patient reports documented responses in bilateral JRHs and a giant RH with extrascleral extension [3, 14–17].

Accordingly, the present study does not seek to establish novelty through cohort size or the observation of ocular response itself. Rather, its contribution lies in methodologically structured longitudinal image analysis, including triplicate pixel-based measurements by three retinal specialists masked to one another’s measurements, restriction to native unmodified fundus images, predefined separation of the largest baseline lesion per eye from exploratory all-lesion analyses, and explicit reporting at the patient, eye, and lesion levels. Methodologically structured quantitative evidence from routine clinical practice nevertheless remains limited.

This retrospective observational study evaluates ocular outcomes and observed tolerability during oral belzutifan treatment at a starting dose of 120 mg once daily in a consecutive real-world cohort of patients with genetically confirmed VHL disease and eyes containing at least one RH that had progressed despite prior conventional local ocular therapy. The largest baseline RH in each fundus-imaged eye constituted the predefined primary analysis, while all fundus-imaged RHs were assessed exploratorily. Exploratory overall ocular response (OOR) and other ocular outcomes were evaluated separately at the eye level, whereas on-treatment follow-up (FU), treatment modifications, and tolerability were assessed at the patient level.

## Materials and methods

### Study design and ethics

This retrospective, single-center observational study was conducted at the Department of Ophthalmology, Ulm University Hospital. The study was conducted in accordance with the Declaration of Helsinki. Based on the submitted project information, the local Ethics Committee of Ulm University determined that no formal ethics vote was required for the anonymized project. All patients had provided written informed consent for the use of clinical data and imaging for research purposes. No reference number was assigned.

### Participants and units of analysis

Five consecutive adults with genetically confirmed VHL disease who initiated oral belzutifan between January 2023 and June 2025 were included, with FU data available through January 2026. Seven eyes were eligible; each eye had at least one RH with documented progression despite prior conventional local ocular therapy. Progression was defined as documented lesion enlargement and/or worsening RH-associated exudative activity on serial clinical assessment or imaging. Eligibility and prior treatment were defined at the eye level and did not imply that every individual lesion had been directly treated. Four eyes had received intravitreal anti-VEGF treatment and four eyes had received focal laser treatment, with one eye represented in both categories; thus, all seven study eyes had undergone prior ocular treatment (Table 2).

Variables were analyzed and reported according to the level at which they were measured. Demographic and systemic characteristics, treatment indication, treatment exposure, treatment modifications, and adverse events (AEs) were assessed at the patient level (Table 1). Best-corrected visual acuity (BCVA), central subfield thickness (CST), OCT biomarkers, choroidal thickness, exudative retinal detachment (RD), and OOR were assessed at the eye level (Table 2). Tumor regression was assessed at the lesion level (Tables 3 and 4). Outcome-specific denominators and the number of evaluable observations were reported throughout.

**Table 1:** Patient-level characteristics, on-treatment fu and observed tolerability during belzutifan treatment (n = 5 patients)

Characteristics	Belzutifan (n = 5)
Age (years), mean ± SD	38.6 ± 13.5
Sex, n (%)	
- male / female	4 (80%) / 1 (20%)
**On-treatment FU (weeks), median (IQR)**	43 (27 - 50)
**Initial dose**	120 mg daily
**Documented cumulative belzutifan exposure (weeks), median (range)**	43 (27 - 50)
**Treatment modifications, n (%)**	2 (40%)
- Dose reduction, n (%)	0 (0%)
- Temporary treatment interruption, n (%); duration	1 (20%); 4 weeks
- Permanent treatment discontinuation, n (%); timing	1 (20%); week 27
**Drug-related AE, n (%)**	
- anaemia	4 (80%)
- fatigue	3 (60%)
- hypoxia	2 (40%)
**Oncological event, n (%)**	
- Metastatic malignant neoplasm progression	1 (20%)
**VHL-associated systemic disease, n (%)**	
- CNS	4 (80%)
- PCC	0
- pNET	0
- RCC	1 (20%)
**Treatment indication for belzutifan, n (%)**	
- CNS	1 (20%)
- RH	3 (60%)
- RCC	1 (20%)

On-treatment FU was defined as calendar time from the first dose to permanent discontinuation or the last on-treatment visit and included temporary interruptions. Documented cumulative belzutifan exposure was calculated by subtracting documented temporary interruption periods. One patient had a single 4-week temporary interruption and subsequently resumed treatment; a different patient permanently discontinued treatment at week 27 following metastatic RCC progression and a change in oncological treatment. No post-discontinuation follow-up was included

Individual patient-level exposure values are not displayed to minimize the risk of re-identification; exposure is summarized using aggregate statistics and the observed range

Abbreviations: AE: adverse event; CNS: central nervous system hemangioblastoma; FU: follow-up; IQR: interquartile range; PCC: pheochromocytoma

pNET: pancreatic neuroendocrine tumour; RCC: renal cell carcinoma; RH: retinal hemangioblastoma; SD: standard deviation; VHL: von Hippel-Lindau disease

**Table 2:** Eye-level characteristics and outcomes during Belzutifan Treatment (n = 7 eligible Eyes)

**Total eyes with RH**,** n**		**7**
**Prior ocular treatments per eye (** ***n*** ** = 7 eligible eyes)**		
− IVT, n (%)		4 (57.1%)
− Focal laser, n (%)		4 (57.1%)
− Other *		0
**Eye ID**	**Prior ocular treatment (weeks before belzutifan initiation)**	
1	Focal laser (25)	
2	IVT (9)	
3	IVT (4)	
4	Focal laser (22)	
5	Focal laser (26)	
6	IVT (8)	
7	Focal laser (NR) / IVT (122)	
**OOR per eye ‡(n = 6 evaluable eyes), n (%)**		
− Improved	6 (100%)	
− Stable	0	
− Worsened	0	
**Functional and Anatomical Outcomes ‡(n = 6 evaluable Eyes)**	Baseline Visit	Last On-Treatment FU Visit
BCVA (LogMAR), median (IQR)	0.8 (0.00 - 1.13)	0.5 (0.00 - 1.13)
CST (μm), median (IQR)	319 (263 - 1048)	280 (221 - 626)
Presence of OCT biomarkers, n		
− IRF : SRF : HRF	4 : 4 : 4	3 : 1 : 2
Choroidal thickness (μm), median (IQR)	460 (420 - 539)	433 (381 - 456)
RH-associated exudative RD, n (%)	4 (66.7%)	1 (16.7%)

Eye IDs are independently assigned, table-specific presentation codes without analytical meaning. They neither encode patient identity or laterality nor correspond to identifiers used in other tables. One eye had received both focal laser and IVT

Eyes may have undergone repeated focal laser treatment and/or received multiple IVTs. For each treatment modality, values in parentheses indicate the interval, in weeks, between the most recent treatment and belzutifan initiation

‡ One eye was not evaluable for BCVA, CST, OCT-biomarker, choroidal-thickness, or OOR analyses because phthisis bulbi and optical media opacity precluded reliable functional assessment and comparable longitudinal fundus and OCT imaging

Other *: cryotherapy, external beam radiation, photodynamic therapy or pars plana vitrectomy.

Abbreviations: BCVA: best-corrected visual acuity; CST: central subfield thickness; FU: follow-up; HRF: hyper-reflective foci; ID: identifiers; IRF: intraretinal fluid; IQR: interquartile range; IVT: intravitreal treatment with anti-vascular endothelial growth factor; JRH: juxtapapillary retinal hemangioblastoma; NR: not retrievable from the retrospective medical records; OOR: Overall ocular response; RD: retinal detachment; RH: retinal hemangioblastoma; PRH: peripheral retinal hemangioblastoma; SRF: subretinal fluid

**Table 3:** Lesion-level characteristics and outcomes during belzutifan treatment (n = 14 fundus-imaged RHs and 1 MRI-assessed RH)

Total number of RH, n	15
- Fundus-imaged RHs assessed for tumor size regression, n	14
- MRI-assessed RH reported separately*, n	1*
**Location of fundus-imaged RH (n = 14), n (%)**	
- JRH / PRH	2 (14.3%) / 12 (85.7%)
**Number of RH per eye, median (IQR)**	2 (1 - 2)
Tumor size regression of largest RH per eye*‡ (6 lesions in 6 eyes from 4 patients) (%), median (IQR)	58.3 (17.2 - 64.7)
**Index lesions showing regression, n/N (%)**	6/6 (100%)
Overall tumor size regression* (%), median (IQR)	57.2 (28.5 - 66.9)
- Tumor size regression > 20%, n/N (%)	11/14 (78.6%)

* One eye was not evaluable for fundus-based lesion-size analysis because phthisis bulbi and optical media opacity precluded comparable longitudinal fundus imaging; the RH in this eye was assessed separately by MRI

‡ In a patient-level sensitivity analysis including the single largest baseline fundus-imaged RH per patient, all four lesions regressed, with a median projected pixel area reduction of 59.6% (range, 14.5 - 64.8%)

Abbreviations: IQR: interquartile range; JRH: juxtapapillary retinal hemangioblastoma; MRI: magnetic resonance imaging;RH: retinal hemangioblastoma; PRH: peripheral retinal hemangioblastoma.

**Table 4:** Individual RH size changes during belzutifan treatment (14 fundus-imaged RHs; 1 MRI-assessed RH reported separately)

RH ID	Measurement Modality	Baseline projected Pixel Area	Last FU projected Pixel Area	Regression (%)	Regression >20%
**RH1**	**Fundus imaging**	**3,005**	**2,460**	**18.1**	**No**
RH2	Fundus imaging	359	168	53.2	Yes
**RH3**	**Fundus imaging**	**60,513**	**21,355**	**64.7**	**Yes**
**RH4**	**Fundus imaging**	**17,019**	**7,762**	**54.4**	**Yes**
**RH5**	**Fundus imaging**	**1,034**	**390**	**62.3**	**Yes**
RH6	Fundus imaging	476	34	92.9	Yes
RH7	Fundus imaging	280	15	94.6	Yes
RH8	Fundus imaging	1,149	307	73.3	Yes
**RH9**	**Fundus imaging**	**3,398**	**1,195**	**64.8**	**Yes**
RH10	Fundus imaging	662	655	1.1	No
RH11	Fundus imaging	624	249	60.1	Yes
RH12	Fundus imaging	2,530	1,706	32.6	Yes
**RH13**	**Fundus imaging**	**11,858**	**10,137**	**14.5**	**No**
RH14	Fundus imaging	4,392	2,987	32.0	Yes
RH15*	MRI caliper*	1.9 × 1.8 mm*	1.8 × 1.8 mm*	5.3*	N/A*

RH IDs were assigned solely for presentation and do not reflect patient, eye, treatment, or chronological order

Bold rows indicate the RH with the largest baseline pixel area in each fundus-imaged eye. These six RHs were included in the prespecified primary analysis

For 14 lesions, projected pixel area was quantified on native fundus images.Regression was calculated as (baseline projected pixel area - last on-treatment FU projected pixel area) / baseline projected pixel area × 100

* RH15 was assessed using MRI caliper measurements and is presented descriptively only. Owing to the different imaging modality and measurement scale, it was excluded from the prespecified primary and exploratory fundus-based analyses

Abbreviations: FU: follow-up; ID: identifier; MRI: magnetic resonance imaging; N/A: not applicable; RH: retinal hemangioblastoma

### Interdisciplinary VHL management and systemic surveillance

All patients were managed within an established interdisciplinary VHL surveillance program involving ophthalmology, neurology, oncology/neuro-oncology, radiology, nephrology, and internal medicine. Baseline ophthalmic and systemic assessments, including clinical examination, laboratory testing, and MRI-based whole-body surveillance, were performed shortly before the first belzutifan dose according to the institutional VHL protocol. All patients underwent at least two surveillance visits during the defined on-treatment period.

Treatment decisions, imaging findings, and AEs were reviewed in an interdisciplinary setting, including the institutional neuro-oncology tumor board when applicable.

Drug-related AEs were graded according to the Common Terminology Criteria for Adverse Events (CTCAE), version 5.0 [18]. Treatment relatedness was based on contemporaneous assessment by the treating interdisciplinary team. Hematological parameters and pulse oximetry were monitored regularly during treatment, and treatment modifications were implemented based on tolerability and interdisciplinary safety assessment. Systemic tumor progression, when present, was documented separately as a reason for treatment discontinuation.

### Ophthalmologic assessment and endpoint-specific evaluability

A standardized ophthalmologic examination was performed at baseline, shortly before initiation of belzutifan therapy, and during on-treatment FU at intervals of approximately 1 to 3 months. Each visit included BCVA assessment (Snellen, converted to logMAR), intraocular pressure measurement, slit-lamp biomicroscopy, and dilated fundus examination.

Multimodal imaging, when feasible, comprised widefield color fundus photography (CFP) and red-free imaging (Clarus 700, Carl Zeiss Meditec Inc.), widefield fluorescein angiography (FA) using gaze-steered multifield acquisitions, and spectral-domain optical coherence tomography (SD-OCT) (Heidelberg Spectralis, Heidelberg Engineering GmbH, or ZEISS Cirrus 5000, Carl Zeiss Meditec Inc.). Quantitative tumor area measurements were performed exclusively on native Clarus 700 CFP and red-free images. Fundus imaging and FA were used to assess lesion number, size, topography, feeder-vessel caliber, exudation or hemorrhage, intralesional vascularity, and fibrosis. OCT was used to assess CST, intraretinal fluid (IRF), subretinal fluid (SRF), hyperreflective foci (HRF), and choroidal thickness.

One eye had optical media opacity in the setting of phthisis bulbi, precluding comparable longitudinal fundus and OCT imaging. This eye was therefore excluded from standardized eye-level BCVA, CST, OCT-biomarker, choroidal-thickness, and OOR analyses. Its RH was assessed separately by MRI calipers and retained only as a descriptive lesion-level observation. The corresponding patient remained included in all patient-level analyses of treatment exposure, on-treatment FU, treatment modifications, and safety.

### Quantitative tumor size assessment

Projected pixel area was quantified for 14 RHs in six eyes with comparable artifact-free native CFP or red-free images at baseline and the last on-treatment FU using Fiji/ImageJ software (ImageJ version 1.54r, National Institutes of Health, Bethesda, MD, USA). Baseline and on-treatment FU images for each lesion were acquired with the same imaging device, modality, and acquisition protocol and were analyzed without post-acquisition rescaling, montage reconstruction, composite image generation, or other image modification.

For each lesion and time point, projected pixel area was determined by manual delineation of the lesion margins. Measurements were performed independently by three retinal specialists (A.K., D.V., and A.W.) who were masked to one another’s measurements. The mean of the three reader-specific measurements at each time point was used for analysis. Fundus-based tumor regression was calculated as [(baseline projected pixel area - last on-treatment FU projected pixel area) / baseline projected pixel area] × 100, with positive values indicating regression. Because the analysis used within-lesion ratios rather than absolute comparisons of physical lesion area between eyes or patients, axial length-based scaling correction was not applied.

The largest baseline RH in each fundus-imaged eye constituted the predefined primary analysis set (six lesions in six eyes). This selection reduced multiplicity from several lesions within the same eye but did not remove correlation between fellow eyes from the same patient; the six eyes were contributed by four patients (Tables 3 and 4). All 14 fundus-imaged RHs were additionally assessed in a secondary exploratory descriptive analysis. A patient-level sensitivity analysis used the single largest baseline fundus-imaged RH per patient; four patients contributed comparable projected-pixel-area measurements (Tables 3 and 4). The association between baseline projected pixel area and relative regression was explored descriptively in the primary lesion set.

The additional RH was assessed separately using two orthogonal MRI caliper diameters (baseline, 1.9 × 1.8 mm; last on-treatment FU, 1.8 × 1.8 mm). A modality-specific area proxy was calculated descriptively as the product of these diameters. Because MRI caliper measurements and fundus projected pixel area are not directly interchangeable, this RH was not pooled with either fundus-imaged lesion set (Tables 3 and 4).

Quantitative lesion-size measurement and qualitative OOR grading were conducted as two separate assessments. For quantitative analysis, anonymized fundus images were presented in random order without information on patient identity, treatment course, or image chronology; the three readers were masked to one another’s measurements. OOR grading was performed subsequently as a separate longitudinal assessment for which baseline and follow-up chronology was necessarily visible; readers remained masked to one another’s assessments.

### Eye-level clinical outcome assessment

OOR was assessed as an exploratory, nonvalidated eye-level morphological composite, separately from quantitative projected pixel-area analysis. Three retinal specialists (A.K., D.V., and A.W.) independently compared paired baseline and last on-treatment FU multimodal ophthalmic imaging for each evaluable eye. Readers were masked to one another’s assessments but not to image chronology. Four domains were graded: feeder-vessel caliber, exudate and/or hemorrhage, intralesional vascularity, and fibrosis (Table 5).

**Table 5:** Independent reader grading of Eye-Level OOR during Belzutifan Treatment (6 evaluable Eyes)

Assessment ID	Reader A.K. Total Score	Reader D.V. Total Score	Reader A.W. Total Score	Eye-Level OOR
OOR1	+ 2	+ 3	+ 2	Improved
OOR2	+ 4	+ 3	+ 3	Improved
OOR3	+ 4	+ 4	+ 4	Improved
OOR4	+ 2	+ 3	+ 2	Improved
OOR5	+ 3	+ 2	+ 2	Improved
OOR6	+ 3	+ 3	+ 3	Improved

OOR assessment IDs were assigned independently for this table and do not correspond to patient, eye, treatment, or identifiers used in other tables. One additional eye was not evaluable for OOR and was excluded from reader grading

OOR was assessed independently by three retinal specialists using feeder-vessel caliber, exudate and/or hemorrhage, intralesional vascularity, and fibrosis. Readers were masked to one another’s assessments but not to image chronology

Decrease or resolution in the first three domains and new or increased fibrosis consistent with involution were assigned + 1; no relevant change was assigned 0; opposite change was assigned − 1

Nonassessable domains were omitted. Total scores ≥ + 2 were classified as improved, 0 or + 1 as stable, and < 0 as worsened

All 18 reader-level classifications in the six evaluable eyes were improved (100% raw agreement); Fleiss’ kappa was not estimable because all ratings were confined to one category

Abbreviations: ID: identifiers; NA: not available; NE: not evaluable; OOR: overall ocular response

A decrease in feeder-vessel caliber, a decrease or resolution of exudate and/or hemorrhage, a decrease in intralesional vascularity, and development or increase of fibrosis consistent with lesion involution were each assigned + 1. No relevant change was assigned 0, and a change in the opposite direction was assigned − 1. Nonassessable domains were omitted from the total score. Scores were summed separately for each reader; totals of at least + 2 were classified as improved, totals of 0 or + 1 as stable, and totals below 0 as worsened. Eyes without comparable baseline and on-treatment FU ophthalmic imaging were classified as not evaluable. OCT-derived IRF, SRF, macular edema, and RH-associated exudative RD were evaluated separately and were not included in the OOR score.

### Treatment protocol

Belzutifan was administered orally at a dose of 120 mg once daily. Treatment was intended to continue without a fixed duration while clinically indicated and tolerated; no minimum exposure was prespecified before response assessment. On-treatment FU was defined as the calendar interval from the first dose to the last on-treatment assessment or to the date treatment was stopped and not subsequently resumed. Temporary interruptions followed by treatment resumption were included in this calendar-time interval. Documented cumulative belzutifan exposure was calculated by subtracting all documented periods without dosing (Table 1). If an interruption was not followed by resumption and subsequently resulted in permanent discontinuation, the interruption date was considered the end of on-treatment FU; no later observations were included. Treatment continuation, interruption, dose reduction, or permanent discontinuation was guided by clinical tolerability and interdisciplinary safety assessment and followed routine clinical practice. Local ocular therapies during on-treatment FU were permitted if clinically indicated and were documented.

### Statistical analysis

Statistical analyses were performed using SPSS Statistics version 29.0.1.0 (IBM Corp.). Given the small sample size and hierarchical data structure, outcomes were summarized descriptively. Continuous data are reported as mean ± standard deviation (SD), median with interquartile range (IQR), or median with range, as specified; categorical data are reported as n/N (%).

For the predefined primary anatomical analysis, percentage change in projected pixel area was summarized for the largest baseline fundus-imaged RH per eye (six lesions in six eyes from four patients) using the median and IQR. Because the six eyes were contributed by only four independent patients and fellow-eye correlation could not be accounted for, no formal hypothesis testing was performed. A patient-level sensitivity analysis included the single largest baseline fundus-imaged RH per patient with comparable fundus imaging (four lesions from four patients) and was summarized using the median and range (Table 3). The exploratory all-lesion analysis, the separately MRI-assessed lesion, and other ocular outcomes were also summarized descriptively. No formal sample-size calculation was undertaken.

Reader-specific OOR categories were tabulated, and raw category agreement was reported. Fleiss’ kappa was not estimable because all 18 evaluable reader-level ratings were assigned to the improved category, resulting in no marginal category variance. Potential genotype-response patterns were explored descriptively without inferential testing.

## Results

### Patient-level characteristics and on-treatment FU

Five consecutive patients with genetically confirmed VHL disease were included. The mean age was 38.6 ± 13.5 years, and four patients (80%) were male (Table 1). Four patients presented with CNS hemangioblastomas, and one patient had concomitant renal cell carcinoma (RCC). Median on-treatment FU was 43 weeks (IQR, 27–50). Documented cumulative belzutifan exposure after excluding the single four-week temporary interruption followed by treatment resumption was 43 weeks (range, 27–50) (Table 1).

### Individual tumor regression

Fifteen RHs were detected. Fourteen lesions in six fundus-imaged eyes were longitudinally measurable using projected pixel area, whereas one additional RH was assessed separately using MRI calipers (Tables 3 and 4).

All six index lesions, defined as the largest baseline fundus-imaged RH in each of six eyes from four patients, regressed during belzutifan treatment. The median reduction in projected pixel area was 58.3% (IQR, 17.2–64.7%) (Table 3). In the patient-level sensitivity analysis, all four index lesions likewise regressed, with a median reduction of 59.6% (range, 14.5–64.8%) (Table 3). In the exploratory all-lesion analysis (14 lesions in six eyes), median regression was 57.2% (IQR, 28.5–66.9%), and 11/14 lesions (78.6%) showed > 20% regression (Tables 3 and 4). The separately assessed MRI RH decreased from 1.9 × 1.8 mm at baseline to 1.8 × 1.8 mm at FU, corresponding to a 5.3% decrease in the product of the two orthogonal diameters. This modality-specific observation was not pooled with the fundus-based projected-pixel-area results.

No clear association was observed between baseline projected pixel area and relative tumor regression. On descriptive inspection, no apparent genotype-specific response pattern was observed. Given the limited sample size and heterogeneous variants, genotype-response analyses were considered exploratory.

**Fig. 1:**
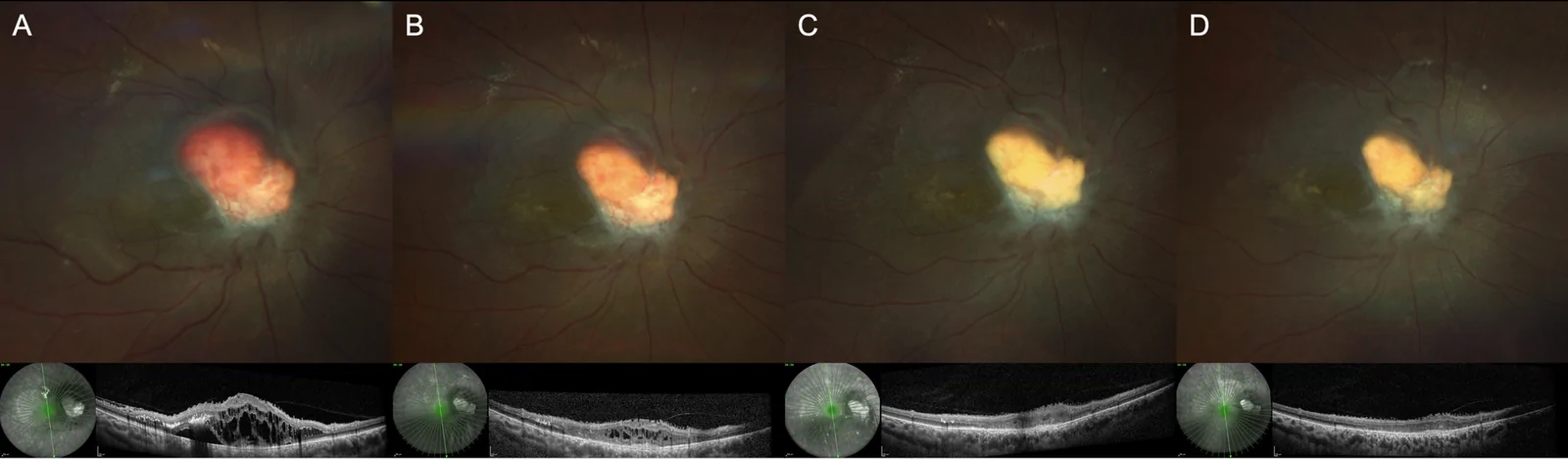
Color fundus photography (CFP) with corresponding spectral-domain optical coherence tomography (SD-OCT) of a patient with a juxtapapillary retinal hemangioblastoma (JRH) that had progressed despite prior intravitreal anti-vascular endothelial growth factor (anti-VEGF) therapy, demonstrating tumor regression and fluid reduction during systemic belzutifan treatment. (**A**) CFP and SD-OCT at baseline before initiation of belzutifan therapy. (**B**) CFP and SD-OCT at week 6 of belzutifan treatment. (**C**) CFP and SD-OCT at week 10 of belzutifan treatment. (**D**) CFP and SD-OCT at week 50, corresponding to the last FU visit, showing tumor size regression of approximately 54% with sustained resolution of associated intraretinal/subretinal fluid

Among the 14 fundus-imaged lesions, 12 (85.7%) were located in the peripheral retina and two were JRHs. In one case with progression despite prior anti-VEGF therapy, approximately 54% tumor regression with sustained exudation control was observed through week 50 (Fig. 1).

### Overall ocular response

All three readers independently classified all six evaluable eyes as improved (6/6; 100%) (Tables 2 and 5). All 18 reader-level classifications were therefore improved, corresponding to 100% raw category agreement. Fleiss’ kappa was not estimable because all ratings were confined to a single category. One eye was not evaluable because phthisis bulbi and optical media opacity precluded comparable longitudinal ophthalmic imaging; its MRI-assessed lesion was reported separately. No evaluable eye was classified as stable or worsened.

### Other eye-level characteristics and anatomical outcomes

All seven eligible eyes had received prior local ocular treatment before belzutifan initiation: four eyes (57.1%) had received intravitreal anti-VEGF therapy and four eyes (57.1%) had undergone focal laser treatment, with one eye represented in both categories (Table 2). Prior treatment was assigned at the eye level and does not imply that every individual lesion had been directly treated.

Among the six eyes with comparable longitudinal ophthalmic imaging, median CST changed from 319 μm (IQR, 263–1048) at baseline to 280 μm (IQR, 221–626) at last FU, and median choroidal thickness changed from 460 μm (IQR, 420–539) to 433 μm (IQR, 381–456). The presence of OCT biomarkers also decreased over time, with reductions in IRF, SRF, and HRF. RH-associated exudative RD was present in four of six evaluable eyes at baseline and resolved without surgical intervention in three of the four affected eyes by the last on-treatment FU visit (Table 2). These structural and anatomical outcomes were summarized descriptively without inferential testing.

### Functional outcomes

Among the six evaluable eyes, median BCVA changed from 0.8 logMAR (IQR, 0.00-1.13) at baseline to 0.5 logMAR (IQR, 0.00-1.13) at last on-treatment FU (Table 2). This functional outcome was summarized descriptively without inferential testing.

### Observed tolerability and treatment modifications

All five treated patients contributed to the patient-level treatment and tolerability analyses. Treatment modifications occurred in 2/5 patients (40%). One patient (20%) had a four-week temporary treatment interruption because of grade 3 anemia and subsequently resumed belzutifan. In a different patient with RCC, belzutifan was withheld at week 27 after metastatic disease progression and was not resumed; for the analysis, this was therefore classified as permanent treatment discontinuation at week 27, and on-treatment FU ended on the date treatment was withheld. Observed drug-related adverse events included anemia in 4/5 patients (80%), fatigue in 3/5 (60%), and hypoxia in 2/5 (40%). Most events were mild to moderate. The oncological event was documented separately from drug-related AEs and led to a change in oncological treatment to adjuvant pembrolizumab following nephrectomy (Table 1).

## Discussion

In this exploratory retrospective cohort of selected eyes containing at least one RH with progression despite prior local ocular therapy, oral belzutifan was associated with anatomical tumor regression and improvement in exudative disease activity. The predefined largest-lesion-per-eye descriptive analysis showed regression in all six index lesions, with a median reduction of 58.3%. In the patient-level sensitivity analysis, all four index lesions regressed, with a median reduction of 59.6%. A similar magnitude of regression was observed across all 14 fundus-imaged lesions. These findings complement the ocular subgroup analysis of LITESPARK-004 and prior real-world reports [3, 13, 14, 16, 17]. The present cohort is neither the first nor the largest to demonstrate ocular response to belzutifan. Jamshidi et al. reported a larger institutional cohort of seven patients and 12 eyes, while Ercanbrack et al. had already provided quantitative Optos-based surface-area measurements in a three-patient case series [3, 16]. Dameron et al. and Cotton et al. further documented responses in bilateral juxtapapillary disease and a giant RH with extrascleral extension, respectively [14, 17]. Accordingly, the point of differentiation of the present study is methodological rather than numerical.

A favorable response was also observed in JRHs, which represent a particular therapeutic challenge due to the risk of collateral retinal or optic nerve damage associated with local ablative therapies near the optic nerve head [7, 10, 11]. Tumor regression and control of exudation under belzutifan highlight the potential of a non-ablative, mechanism-based systemic approach in this high-risk subgroup, in line with recent reports on advanced or anti-VEGF-refractory juxtapapillary lesions [3, 14, 16, 17]. Recent studies have reported favorable outcomes with minimally ablative JRH treatments. Ketkar et al. described anatomical stabilization and SRF improvement in eight eyes treated primarily with indocyanine green-guided transpupillary thermotherapy; however, only five of seven patients had genetically confirmed VHL disease, several eyes received adjunctive intravitreal dexamethasone and/or bevacizumab, and quantitative tumor regression was not assessed, limiting direct comparability with the present study [19].

BCVA showed numerical improvement and was summarized descriptively. Given the exploratory nature of the study, the limited sample size, and chronic structural retinal damage at baseline, marked functional recovery was not expected. Previous reports have similarly described visual stabilization despite substantial anatomical regression, suggesting that visual outcomes may be constrained by irreversible retinal changes rather than ongoing tumor activity alone [5, 13]. In this context, visual stabilization may represent a clinically relevant outcome in advanced or previously treated ocular VHL disease.

The observed morphological response was not confined to projected-pixel-area reduction. The exploratory OOR assessment complemented quantitative lesion measurements by summarizing changes in feeder-vessel caliber, exudate and/or hemorrhage, intralesional vascularity, and fibrosis. OCT-derived CST, IRF, and SRF, as well as RH-associated exudative retinal detachment, were evaluated separately. OOR should nevertheless be interpreted only as a nonvalidated descriptive composite and not as a substitute for the separately reported quantitative and anatomical outcomes.

An important limitation is the hierarchical structure of the data, with multiple lesions nested within eyes and two patients contributing both eyes. Selecting the largest baseline lesion per eye reduced multiplicity arising from several lesions within the same eye but did not eliminate correlation between fellow eyes exposed to the same systemic treatment. Because the primary anatomical analysis comprised six lesions from six eyes contributed by only four independent patients, reliable marginal or mixed-effects modeling and formal patient-level inference were not feasible. The patient-level sensitivity analysis avoided fellow-eye duplication but included only four observations and therefore cannot establish a confirmatory treatment effect. Accordingly, both analyses should be interpreted descriptively and as hypothesis-generating.

Several aspects of the study strengthen the transparency of the observed findings. Tumor regression was evaluated using a predefined index-lesion analysis and an exploratory all-lesion summary. Quantitative tumor area analysis was deliberately restricted to native, standardized, unmodified CFP and red-free images, without image rescaling, montage reconstruction, or composite image generation. Three retinal specialists independently measured each fundus-imaged lesion, and qualitative OOR grading was reported separately at the eye level. Presenting patient-, eye-, and lesion-level outcomes with outcome-specific denominators improves reproducibility, although it cannot remove the underlying within-patient correlation. Furthermore, belzutifan therapy was embedded in a structured interdisciplinary management framework with standardized safety monitoring and CTCAE-based AE grading, enabling reliable tolerability assessment under real-world conditions.

Regarding observed tolerability, the AE profile observed in our cohort was consistent with previously published clinical trial data and real-world reports, with anemia, fatigue, and hypoxia being the most relevant treatment-emergent events [4, 13, 15, 20]. No unexpected ocular safety signals were identified. Nevertheless, belzutifan therapy requires careful contextualization. The drug is currently used off-label for ocular manifestations of VHL disease, is associated with considerable treatment costs, requires structured laboratory and clinical monitoring, and may cause systemic adverse events. Moreover, tumor control may be treatment-dependent, as recurrent RH activity has been reported during dose reduction or treatment interruption [3]. Thus, belzutifan should not be viewed as a universal stand-alone replacement for established local therapies, but rather as a systemic treatment option within an interdisciplinary and individualized treatment concept.

This contextualization is particularly relevant because VHL disease is typically bilateral, multifocal, and multisystemic. Many affected patients have multiple ocular lesions, recurrent or previously treated RHs, and extraocular manifestations requiring lifelong surveillance and interdisciplinary management [1, 21]. In such patients, systemic HIF-2α inhibition may offer the advantage of simultaneously addressing ocular and extraocular disease activity through a shared molecular pathway. The inclusion of pretreated patients reflects this clinical scenario and supports the role of belzutifan as an adjunctive or complementary strategy for selected patients with advanced, refractory, recurrent, or anatomically high-risk ocular VHL disease.

Previous studies, most notably by Chen et al., have demonstrated that ultra-widefield FA, particularly with gaze steering, can substantially increase detection of small and far-peripheral RHs compared with conventional imaging [22]. Accordingly, it cannot be excluded that very peripheral or subclinical lesions may have been underdetected in the present study. However, maximal lesion detection and reproducible longitudinal tumor quantification are not identical objectives. Ultra-widefield and montage-based imaging can be affected by peripheral distortion, uneven illumination, fluorescein leakage, and imaging artefacts, which may impede reliable delineation of tumor margins and longitudinal comparability. This is supported by recent evidence demonstrating potential montage-related errors in ultra-widefield imaging of retinal hemangioblastomas [23]. Therefore, the imaging strategy in the present study was deliberately conservative and focused on reproducible measurement of predefined lesions on native images.

Further limitations include the retrospective design, small cohort size, lack of a control group, and limited FU duration, which limit the precision and generalizability of the descriptive estimates and preclude definitive conclusions regarding long-term durability and functional outcomes. Because OOR grading required comparison of baseline and follow-up images, it was not masked to image chronology, which may have introduced expectation bias into this exploratory qualitative outcome. This limitation does not apply to the separately conducted, chronology-masked quantitative tumor-size analysis. One eye received adjunctive parabulbar triamcinolone during on-treatment FU, which may have influenced exudative, OCT-based, and OOR outcomes independently of belzutifan. Nevertheless, given the rarity of ocular VHL disease, the use of standardized multimodal imaging, quantitative lesion-based analyses, eye-based qualitative response assessment, and structured interdisciplinary management provides valuable real-world evidence complementing data from controlled trials and published case series [3, 13].

## Conclusions

Oral belzutifan was associated with RH regression and improved exudative disease activity in this selected real-world cohort. These findings support systemic HIF-2α inhibition as a potential adjunctive therapeutic option for selected ocular manifestations of VHL disease, particularly in patients with RHs that progressed despite prior local treatment or with multifocal or anatomically high-risk lesions.

Larger prospective studies with extended FU are warranted to confirm durability, refine patient selection, and better define the role of belzutifan in ocular VHL disease. Future investigations may further clarify its role in carefully selected patients with advanced disease and limited remaining visual reserve, including last-seeing-eye situations.

## Data Availability

The data supporting the findings of this study are available from the corresponding author upon reasonable request, subject to applicable data protection requirements.

## Abbreviations

AE: Adverse event
BCVA: Best-corrected visual acuity
CFP: Color fundus photography
CNS: Central nervous system
CST: Central subfield thickness
CTCAE: Common terminology criteria for adverse events
FA: Fluorescein angiography
FU: Follow-up
HGVS: Human genome variation society
HIF: Hypoxia-inducible factor
HRF: Hyperreflective foci
ID: Identifier
IOP: Intraocular pressure
IQR: Interquartile range
IRF: Intraretinal fluid
IVT: Intravitreal anti-VEGF therapy
JRH: Juxtapapillary retinal hemangioblastoma
MLPA: Multiplex ligation-dependent probe amplification
MRI: Magnetic resonance imaging
OOR: Overall ocular response
PRH: Peripheral retinal hemangioblastoma
RCC: Renal cell carcinoma
RD: Retinal detachment
RH: Retinal hemangioblastoma
SD: Standard deviation
SD-OCT: Spectral-domain optical coherence tomography
SRF: Subretinal fluid
VEGF: Vascular endothelial growth factor
